# Application and evaluation of automated methods to extract neuroanatomical connectivity statements from free text

**DOI:** 10.1093/bioinformatics/bts542

**Published:** 2012-09-06

**Authors:** Leon French, Suzanne Lane, Lydia Xu, Celia Siu, Cathy Kwok, Yiqi Chen, Claudia Krebs, Paul Pavlidis

**Affiliations:** ^1^Department of Psychiatry, ^2^Centre for High-Throughput Biology and ^3^Department of Cellular and Physiological Sciences, University of British Columbia, Vancouver, BC, V6T 1Z4, Canada

## Abstract

**Motivation:** Automated annotation of neuroanatomical connectivity statements from the neuroscience literature would enable accessible and large-scale connectivity resources. Unfortunately, the connectivity findings are not formally encoded and occur as natural language text. This hinders aggregation, indexing, searching and integration of the reports. We annotated a set of 1377 abstracts for connectivity relations to facilitate automated extraction of connectivity relationships from neuroscience literature. We tested several baseline measures based on co-occurrence and lexical rules. We compare results from seven machine learning methods adapted from the protein interaction extraction domain that employ part-of-speech, dependency and syntax features.

**Results:** Co-occurrence based methods provided high recall with weak precision. The shallow linguistic kernel recalled 70.1% of the sentence-level connectivity statements at 50.3% precision. Owing to its speed and simplicity, we applied the shallow linguistic kernel to a large set of new abstracts. To evaluate the results, we compared 2688 extracted connections with the Brain Architecture Management System (an existing database of rat connectivity). The extracted connections were connected in the Brain Architecture Management System at a rate of 63.5%, compared with 51.1% for co-occurring brain region pairs. We found that precision increases with the recency and frequency of the extracted relationships.

**Availability and implementation:** The source code, evaluations, documentation and other supplementary materials are available at http://www.chibi.ubc.ca/WhiteText.

**Contact:** paul@chibi.ubc.ca

**Supplementary information:** Supplementary data are available at *Bioinformatics* Online.

## 1 INTRODUCTION

The brain is a vast interconnected network. Each neuron communicates with many others through chemical and electrical synapses to integrate information. Groups of neurons (in structures such as nuclei or layers) make diverse connections across the brain, forming pathways of information flow. This structural connectivity is a major determinant of brain function and is frequently used by neuroscientists and clinicians to interpret physiological data. Examples include understanding strokes (Haines, 2004) and interpreting brain imaging results. Evidence for connectivity abnormalities has been found in bipolar (Houenou *et al.*, 2007), autism (Koshino *et al.*, 2005), Alzheimer’s (Stam *et al.*, 2007) and schizophrenia patients (Karlsgodt *et al.*, 2008). A major goal of modern neuroscience is to understand the organization of the brain at all levels in as much detail as possible, and to understand how this networked organization relates to brain function and ultimately behaviour and human health (Sporns, 2011).

The characterization of the connectivity network or wiring diagram of the brain is incomplete (Crick and Jones, 1993). In part this is due to the complexity of the brain and the difficulty in collecting data. However, we suggest that informatics technologies can be used to leverage existing knowledge that has already been collected to make new discoveries and guide further experimentation.

In this work, we are primarily concerned with ‘macroconnections’, or connections that can be identified between small brain regions (as opposed to microcircuitry, which describes the connections among neurons *per se*). These macroconnections between groups of neurons are predicted to number between 25 000 and 100 000 (Bota *et al.*, 2003). This suggests a high level of complexity, although comfortably placed between the more gross levels of brain organization and the microarchitecture, which encompasses billions of neurons and quadrillions of synapses (Sporns *et al.*, 2005). Furthermore, this estimated number of macroconnections is smaller in scale than estimates of the human protein interactome at 650 000 interactions among 25 000 proteins (Stumpf *et al.*, 2008).

Connectivity between brain regions can be assayed using tract tracing or electrophysiology. Tract tracing typically involves injecting a dye or other tracer (e.g. horseradish peroxidase) into one brain region and tracking the fate of the tracer as it follows axonal pathways (Lanciego and Wouterlood, 2011). Electrophysiological methods use electrical or other stimulation in one site along with electrical recording at a second site to test the functional connectivity of regions. Using these methods, a researcher can determine connections that send signals to the region (afferent) or away from the region (efferent). Over many years, thousands of connectivity studies have been performed, each of which typically elucidates, at most, a few connections. The presence of a deep literature on neuronal connectivity is a major motivation for this work: the data are out there, they just need to be assembled.

Attempts to turn this huge accumulation of knowledge into an ‘omics’ scale database have been limited, despite the potential value of such a resource. Previous efforts have primarily used manual reviews of the literature to laboriously generate connectivity maps for limited parts of the brain. In 1991, Felleman and Van Essen published a connectivity matrix of the macaque visual cortex covering 305 pathways between 32 areas (Felleman and Van Essen, 1991). Currently, a large number of collated connections are stored in the Collations of Connectivity data on the Macaque brain database (CoCoMac) (Kotter, 2004). CoCoMac contains detailed information from 413 literature reports regarding 7007 macaque brain regions. A fourth model organism with large-scale connectivity data is the rat, with more than 40 000 formalized reports of connections in the Brain Architecture Management System (BAMS) (Bota *et al.*, 2005). Information is added to these databases manually, and therefore, they are accurate but sparse. Currently, the only complete connectome scale database is the neuron-level wiring diagram of *Caenorhabditis elegans*, determined from electron micrographs (White *et al.*, 1986).

We sought to extend and complement manual efforts with automated text mining techniques. More than 10 years of efforts to recognize gene and protein mentions and their interactions inspire our work (Blaschke *et al.*, 1999; Jensen *et al.*, 2006). In the gene interaction task, one must extract information from sentences such as ‘gene A interacts with gene B’ (to give a toy example). Despite the difficulty of this task, great progress has been made. A comprehensive evaluation of kernel methods for extracting protein–protein interactions detailed precision and recall values ranging from 45 to 70% by varying experiment design, dataset and method tested (Tikk *et al.*, 2010). At the second Critical Assessment of Information Extraction systems in Biology (BioCreAtIvE II), the top team was able to extract normalized directed interaction pairs from full-text articles, with precision of 37% and recall of 33% (Krallinger *et al.*, 2008). The analogy to brain connectivity is tight: we wish to extract information from sentences akin to ‘brain region A connects to brain region B’. This related research gives us hope that the approaches applied to extracting gene interaction information can successfully mine connectivity relations.

To our knowledge, there have been no previous attempts to extract connectivity information using text mining methods. The closest work to our own is that of Burns *et al.* (2007), which was aimed at extracting information about tract-tracing experiments, trained and evaluated with a manually annotated corpus of 1047 sentences from 21 documents. Although Burns *et al.* describe named entity recognition (e.g. identification of label injection sites), they did not report extraction of connectivity statements themselves.

For the work presented here, we have simplified the problem by limiting our input dataset and output results. We focus on abstracts from one journal, the Journal of Comparative Neurology (JCN), because it is enriched for tract-tracing studies. We used abstracts rather than full-text documents because they are enriched for high-level summary statements and are more accessible. We also break our task into several subtasks, isolating the steps of brain region term recognition and normalization from the evaluations (French *et al.*, 2009; French and Pavlidis, 2011). We only consider the presence of connectivity relations, ignoring the type, strength or direction of the connection. These limitations make the task simpler and set the stage for future more detailed studies.

Our results show that text mining approaches previously used to analyse protein networks can be usefully applied to brain connectivity. Our large manually annotated corpus allowed testing and training of various techniques possible, and we also perform extensive manual validation of the results. Beyond the corpus-based evaluations, we compared a large set of automatically extracted connectivity statements with an existing connectivity database with favourable results. Together with our previous work on term recognition and normalization, we present a completely automated system for extraction of brain connectivity information from abstracts.

## 2 METHODS

### 2.1 Annotated data

To train and test text mining algorithms, we created a large gold standard dataset. This dataset or corpus consists of abstracts manually annotated by an undergraduate research assistant (S.L.) for connection verbs, species of study, brain region mentions and connections between them. We annotated 1377 abstracts for 4276 connections and 17 585 brain region mentions. Abstracts were randomly chosen from the JCN (years 1975–2008). This dataset has been previously used to demonstrate automated brain region recognition and normalization (French *et al.*, 2009; French and Pavlidis, 2011), without using the connectivity annotations. Each annotated connection consists of two brain regions, text describing the connection and the associated organism. This corpus provides sufficient training examples for machine-learning methods.

We developed guidelines and software for the annotation process. Briefly, our main guidelines were as follows: (1) annotate all brain region mentions, regardless of whether they are part of a connection; (2) annotate all connections and brain regions for all organisms and organism states; (3) do not annotate mentions of white matter tracts or nerves; and (4) only annotate monosynaptic or direct connections. We accepted connections that were stated in titles or introductory sentences that assume connections between two high-level regions. Example relationships that were rejected are ‘may be connected’, ‘influences’, ‘invaded’ and ‘alters activity’. The General Architecture for Text Engineering was used by annotators to highlight and connect brain region mentions in text (Cunningham *et al.*, 2002).

### 2.2 Co-occurrence and rule-based methods

To extract neuroanatomical connections as described by the abstract authors, we must at least link two brain region mentions. Our first method, acting as a naïve baseline method, predicts a stated connection between every pair of brain region mentions (Jensen *et al.*, 2006). We evaluate co-occurrence for single sentences and entire abstracts (including title).

We created two simple rule-based extensions of the co-occurrence technique. The first simply limits co-occurrence extraction to sentences with less than a set number of brain region mentions. The second requires presence of a connectivity-related keyword (‘afferent’, ‘efferent’, ‘projects’, ‘projection’, ‘pathway’ or ‘inputs’).

### 2.3 Kernel-based methods

Seven advanced kernel-based methods were applied to the dataset. These methods were originally designed for extraction of protein–protein interactions. Each technique uses different features, parameters and kernel functions. Implementations were brought into a common evaluation framework by Tikk and colleagues (Tikk *et al.*, 2010). Syntax and dependency trees for the sentences were computed by the Charniak-Lease re-ranking (McClosky *et al.*, 2006) and Stanford (De Marneffe *et al.*, 2006) parsers, respectively (same versions used in the Tikk *et al.* framework). The methods are categorized according to the type of features extracted. Four syntax tree-based methods use different techniques to compare the sentence parse trees (Collins and Duffy, 2001; Kuboyama *et al.*, 2007; Moschitti, 2005; Vishwanathan and Smola, 2002). Going beyond syntax parsers, the all-paths graph kernel (Airola *et al.*, 2008) and k-band shortest path spectrum kernel (Tikk *et al.*, 2010) use dependency parse information. Finally, the shallow linguistic kernel (SLK) uses only shallow parsing information such as word occurrences and part-of-speech tags (Giuliano *et al.*, 2006). Further details about the kernels are available on the supplement website. We used this framework to benchmark each of the kernel-based methods on the brain region connectivity task. Of the nine methods described by Tikk *et al.*, we were able to successfully test seven, including the three top-performing kernels reported by Tikk *et al.* (2010). The same parameter sets used by Tikk and colleagues were tested on our corpus.

### 2.4 Experiment setup

We evaluate connection extraction independently of the previously described methods for automated brain region recognition (French *et al.*, 2009). This is done by providing the manually annotated brain region mentions to the relation extraction algorithm. Under this design, the extraction task only requires correct linking of brain region mentions.

To find a high-performing method, the different methods and varying parameters were run on a subset of 1146 abstracts. The top-performing classifier and parameter set were retested on the full set of 1377 abstracts to gauge generalizability. Results for the kernel methods were computed using 10-fold cross-validation. Each sentence became an input instance for the kernel methods (including article title). Sentences of an abstract were not split between training and testing sets (document-level split).

### 2.5 Evaluation

Several evaluations were performed to judge the accuracy of the extracted connectivity statements. We primarily report the results from the cross-validation experiments that test predictions against the manually annotated connections. Detailed evaluation and annotation guidelines are provided as Supplementary Materials.

Performance is measured against the number of true connectivity relations that are annotated completely within a sentence or an abstract. The rule- and co-occurrence-based methods can operate at the abstract or sentence level, whereas the kernel methods are limited to single sentences. Precision is computed as the proportion of predicted relations that are correct, and recall is the proportion of true relations that are predicted by the method. The f-measure is the harmonic mean of these two values, providing a balance of both. We also compute the area under the receiver operating curve where applicable (AUC). This measure uses a ranked list of predictions with descending classification prediction scores that approximate confidence in the prediction. This ranking allows computation of the true-positive and false-positive rates for a range of discrimination thresholds. Previous experiments have found the AUC measure to be more robust and stable than f-measure for interaction mining (Tikk *et al.*, 2010).

### 2.6 Comparison with existing connectivity database

Normalization of brain region mentions to brain region concepts in formalized lexicons was targeted to the BAMS atlas (Swanson, 1999). BAMS was chosen because of its wealth of curated rat-tract-tracing studies (Bota *et al.*, 2005). In addition, rat is the most commonly studied species in our corpus. Our previously described Bag of Stems resolver was applied, with all mention editors used, including those that map region mentions to larger enclosing brain regions (French and Pavlidis, 2011). The lexical information in BAMS was expanded with synonym information to increase normalization performance. All possible normalized parings are evaluated when a mention maps to more than one region. Connections in the BAMS connectivity matrices were up-propagated in the anatomy hierarchy, which ensures that if there is a connection between regions A and B, then all enclosing regions of A and B are also connected. Self-connections extracted from literature were ignored. The Linnaeus species tagger was used to recognize species names in the abstracts (Gerner *et al.*, 2010).

## 3 RESULTS

Our gold standard is a set of manually annotated 4276 brain region connectivity relations across a corpus of 1377 abstracts. To gauge interannotator agreement, a second curator (L.X.) annotated a random subset of 231 documents. Roughly 80% of the second curator’s annotations matched the primary curator (79.5% recall at 82.3% precision). Unlike the automated methods that predict relations between given brain region mention spans, this evaluation required both annotators to mark the same spans and relationships. By removing this restriction and allowing partially matching spans, the precision and recall reach 93.9% and 91.9%, respectively.

We used a co-occurrence analysis to reveal the proportion of brain region mention pairs that are co-mentioned and described as connected. Co-occurrence assumes the relation is a connectivity statement. At the abstract level, this yields a precision of only 2.2% at 100% recall and a f-measure of 4.3%. Within a sentence, co-occurrences between all pairs predict connected pairs at 13.3% precision and 72.4% recall (the remaining relations span sentences). This level of recall means that more than ¼ of all annotated connectivity relations are formed with regions in different sentences. Owing to the difficulty in extracting connections spanning sentences, all of our subsequent evaluations are performed at the sentence level, with the relations spanning sentences excluded. Under this evaluation framework, sentence-level co-occurrence recalls 100% of the remaining 3097 relations.

We tested two simple modifications of the sentence-level co-occurrence technique. The first reduces co-occurrence predictions to sentences with a limited number of brain region mentions. By extracting co-occurring pairs from sentences with only two brain region mentions, precision reaches 23.1% and 17.2% recall (f-measure = 19.7%). This means that an average sentence with two brain region mentions is reporting a connection in almost one of four cases. By varying this threshold, the f-measure increases until sentences with six or more brain region mentions are included. We observed that some of these larger sentences merely list brain regions involved in the study and not their relationships. By limiting the threshold at five brain region mentions or less per sentence, co-occurrence provides 18.8% precision and 66.1% recall (Table 1). The second rule tested requires the sentences contain connectivity-related keywords (see Methods section). This keyword-based rule increases recall to 17.4% and precision to 92.7% (f-measure = 29.4%). We created a new approach named ‘Keyword 5-threshold’ by combining these two rules. This again provides improvement, with f-measure reaching 34.1%. As expected, rule-based methods increase precision at the cost of lower recall when compared with unrestricted co-occurrence.

**Table 1.** Sentence level training set cross-validation results

Kernel	Parser type	Parameter sets	Precision	Recall	F-measure	AUC
Co-occurrence	None	1	13.30%	100.00%	23.50%	
Subset tree kernel	Syntax	12	44.20%	20.80%	28.10%	74.80%
Co-occurrence five threshold	None	25	18.80%	66.10%	29.30%	
Partial tree kernel	Syntax	12	43.30%	23.10%	29.80%	75.20%
Keyword co-occurrence	None	1	17.40%	92.70%	29.40%	
Spectrum tree kernel	Syntax	21	37.40%	26.10%	30.20%	72.90%
Subtree kernel	Syntax	12	40.70%	25.20%	30.80%	74.60%
Keyword five threshold	None	25	23.70%	60.80%	34.10%	
k-band shortest path spectrum	Dependency	288	46.80%	70.50%	55.80%	86.70%
Shallow linguistic kernel (SLK)	Part-of-speech tagger	1	50.30%	70.10%	58.30%	88.90%
All-paths graph kernel	Dependency	4	60.40%	57.90%	58.40%	88.40%

AUC, area under the receiver operating curve; SLK, shallow linguistic kernel.

Next, we applied seven previously published methods for extracting protein–protein interactions to our connectivity relation dataset. Although the methods were designed for a different type of biomedical relation, they did not require any modification for our application. The cross-validation results on the training dataset (1146 abstracts) are provided in Table 1. For each method, the parameter set with the highest AUC score is shown. The parameter sets range in size and were reproduced from Tikk *et al.* without modification (primarily grid searches of support vector machine settings). The f-measure scores for all of the seven methods outperform unrestricted co-occurrence-based analysis for at least one parameter set. The simple rule-based methods outperform the more complex partial tree- and subset tree-based methods. Although all of the syntax tree-based methods are outperformed by the Keyword 5-threshold approach, they provide much higher precision than recall. When ranked by AUC, the SLK performs best with a 58.3% f-measure and an AUC of 88.9%. The All Paths Graph and k-band shortest path spectrum kernel methods rank a close second and third, respectively, with similar scores.

We choose the SLK method for subsequent experiments owing to its accuracy, speed and single parameter set (global n-gram = 3 and local window = 2). Unlike the other kernel methods, the SLK method uses only shallow linguistic information at the local (neighbouring words) and global sentence levels to predict relationships (Giuliano *et al.*, 2006). This information forms feature vectors that are used to train a support vector machine classifier (scalar product kernel). The performance of SLK on the complete set of 1377 abstracts is consistent with the cross-validation results (f-measure of 0.592). Figure S1 displays the resulting receiver operating characteristic (ROC) curve (AUC = 0.899).

**Fig. 1.**
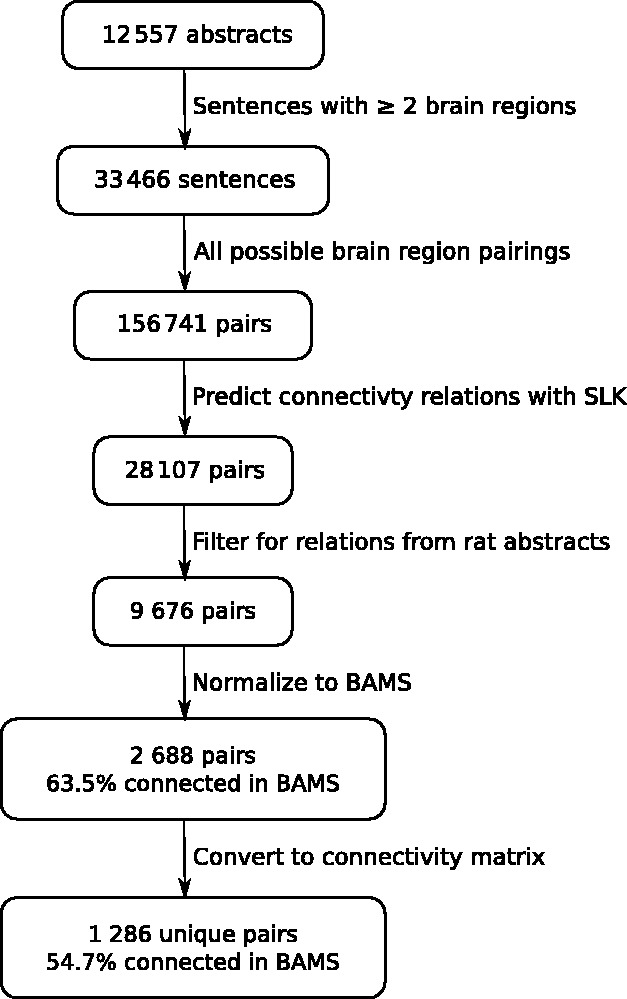
Flow chart depicting the processing steps for comparison with the Brain Architecture Management System

We applied the SLK classifier to candidate sentences extracted from a set of 12 557 abstracts from the JCN (covering 1975–2011), previously unseen by the algorithm. Our automatic brain region recognizer provided 33 466 sentences that mention two or more brain regions (French *et al.*, 2009; French and Pavlidis, 2011). Within these sentences, SLK predicted 18% of the 156 484 possible brain region pairings to be connectivity relations. Of these predicted relations, 9676 are in an abstract that mentions rat and can be evaluated against BAMS. Figure 1 shows the progression from abstracts to predicted connectivity relationships.

To evaluate the precision of the predicted connections, we manually reviewed a random subset of 2000 abstracts. Each pair was evaluated by two curators, yielding an interannotator agreement rate of 85%. Conflicts were resolved by a third curator or by consensus after discussion. Overall, the SLK predictions were 55.3% precise. Errors from the automated steps of named entity recognition and abbreviation expansion were 11% and 4%, respectively. These rates suggest a lesser impact of named entity recognition errors when compared with assessments in the protein–protein interaction domain (Kabiljo *et al.*, 2009). Table 2 presents the five most and least confident connectivity relations for the rat brain. Classification confidence is approximated with the SLK prediction score (distance to classifying hyperplane), with highest values representing the cases closest to positive training examples. Two of the most confident predictions are extracted from an article title and have the same form (ranks 1 and 5). The sentences containing top predictions are shorter on average (192 characters) than the sentences with least confident predictions (282 characters), suggesting sentence complexity affects the prediction results. Of these 10 examples, only one is clearly a false-positive prediction (rank 9764), while several others point to errors in previous automated steps. The mentions of ‘internal capsule’ (rank 9766) and ‘Met-enkephalin’ (rank 4) are incorrectly predicted as brain region mentions (our definition of a brain region excludes fibre tracts like the internal capsule, while enkephalin is a peptide). We manually compared these 10 results with the BAMS system and found it surprisingly difficult to map the mentioned regions to those in BAMS. For example, ‘retrosplenial dysgranular cortex’ and ‘dorsal medullary reticular column’ were not found in BAMS. In the end, corresponding connections were found in BAMS for several of the relationships, but only between enclosing regions (ranks 9767 and 5).

**Table 2.** Top- and bottom-predicted relations from the 12 557 abstract set, ranked by SLK classification score

Rank	Sentence	Score	Reference
1	**Trigeminal** projections to **hypoglossal and facial motor nuclei** in the rat.	3.47	Pinganaud, *et al.*, 1999
2	**The cortical** projections to **retrosplenial dysgranular cortex** (Rdg) originate primarily in the infraradiata, retrosplenial, postsubicular and areas 17 and 18b cortices.	3.34	van Groen and Wyss, 1992
3	The **thalamic** projections to **retrosplenial dysgranular cortex** (Rdg) originate in the anterior (primarily the anteromedial), lateral (primarily the laterodorsal) and reuniens nuclei.	3.33	van Groen and Wyss, 1992
4	Our results indicate that the **centromedial amygdala** receives **Met-enkephalin** afferents, as indicated by the presence of mu-opioid receptor, delta-opioid receptor and Met-enkephalin fibres in the CEA and MEA, originating primarily from the bed nucleus of the stria terminalis and from other amygdaloid nuclei.	3.32	Poulin, *et al.*, 2006
5	**Thalamic** projections to **retrosplenial cortex** in the rat.	3.28	Sripanidkulchai and Wyss, 1986
…	9757 relationships		
9763	The sparse reciprocal connections to the other **amygdaloid nuclei** suggest that the CEA nucleus does not regulate the other amygdaloid regions, but rather executes the responses evoked by the other amygdaloid nuclei that innervate the **CEA nucleus**.	5.46 × 10^−4^	Jolkkonen and Pitkanen, 1998
9764	The majority of the endomorphin 1/fluoro-gold and endomorphin 2/fluoro-gold double-labelled neurons in the **hypothalamus** were distributed in the **dorsomedial nucleus**, areas between the dorsomedial and ventromedial nucleus and arcuate nucleus; a few were also seen in the ventromedial, periventricular and posterior nucleus.	4.36 × 10^−4^	Chen, *et al.*, 2008
9765	Projections from the **dorsal medullary reticular column** are largely bilateral and are distributed preferentially to the **ventral subdivision of the fifth cranial nerve motor nuclei in the rat (MoV)**, to the dorsal and intermediate subdivisions of VII and to both the dorsal and the ventral subdivision of XII.	2.91 × 10^−4^	Cunningham and Sawchenko, 2000
9766	Two additional large projections leave the MEA forebrain bundle in the hypothalamus; the ansa peduncularis–ventral amygdaloid bundle system turns laterally through the **internal capsule** into the striatal complex, **amygdala** and the external capsule to reach lateral and posterior cortex, and another system of fibers turns medially to innervate MEA hypothalamus and median eminence and forms a contralateral projection through the supraoptic commissures.	2.87 × 10^−4^	Moore, *et al.*, 1978
9767	In animals with injected horseradish peroxidase confined within the main bulb, perikarya retrogradely labelled with the protein in the **ipsilateral forebrain** were observed in the **anterior prepyriform cortex horizontal limb of the nucleus of the diagonal band**, and far lateral preoptic and rostral lateral hypothalamic areas.	3.36 × 10^−5^	Broadwell and Jacobowitz, 1976

CEA, central; MEA, medial; MoV, the fifth cranial nerve motor nuclei in the rat.

Encouraged by these results, we did a comparison of the results from the 12 557 abstracts with BAMS, to gauge accuracy of connections and the extent to which our approach might supplement manual curation efforts. Compared with the manual annotations, this is a less precise evaluation because BAMS does not cover the complete literature and is limited to rat studies (Bota *et al.*, 2005). In addition, mapping errors resulting from linking brain region mentions to target regions in BAMS reduces accuracy (French and Pavlidis, 2011). For example, 12% of mentions are mapped to more than one brain region owing to ambiguous synonyms. To benchmark the BAMS evaluation metric, we first tested it on the manually curated connectivity relations from our training corpus of 1377 abstracts. Our process first extracts abstracts that used rat (based on Linneaus analysis) and maps the brain region mentions to the BAMS lexicon. These rat connectivity relationships were then compared with the BAMS connectivity matrix. Only 167 manually annotated connectivity relations were testable by this method, with 70.5% having a connection in BAMS. In the same set of abstracts, the 2617 brain region pairings not annotated as connections but co-occur in sentences are connected in BAMS at 49.8%. This is not surprising because co-occurring regions may be connected, but the author is not stating that in the sentence. In the larger set of rat-related abstracts, 2688 computationally predicted connectivity statements are successfully resolved, and 63.5% are, in fact, reported as true by BAMS (Fig. 1). For comparison, the remaining set of co-occurring brain region pairs is connected in BAMS at a rate of 51.1%. We noted that the extracted relationships are between larger or less specific brain regions than those in BAMS. Anatomical depth, or the average number of enclosing or parent brain regions for a connected pair in the BAMS matrix, is 9.6, whereas the literature-extracted connections had a mean of 7.9, indicating they are larger and less specific brain regions. Along the same lines, the literature-based relationships only involve 433 regions, whereas BAMS has connection reports for 633 regions. We evaluated the 899 connections that are predicted by our method but not listed as connected in BAMS (provided in Supplement). Similar to the previous evaluation of 2000 connections, approximately one-half of these connections are false-positive text mining errors (52.1% precision). The remaining 468 connections that are true positives at the sentence level could be used to expand BAMS coverage (although curation guidelines differ). Within these 468 true positives, we selected a subset of 250 for further review by a domain expert (C. Krebs). Only nine of the predicted connections were rejected (3.6%). Five were rejected because a protein (pituitary adenylate cyclise-activating polypeptide) was incorrectly recognized as a brain region (pituitary gland). This agreement between the curators and an expert suggests our annotation guidelines are consistent and accurate.

We hypothesized that owing to improvements in tracing methods, more recent reports of connectivity would be of higher quality. This was suggested by a study of different eras of tract-tracing techniques that revealed large improvements in accuracy (Bota *et al.*, 2003). Bota and colleagues found that limbic system connections observed using an old method, axon degeneration (Nauta, 1952), are 60% accurate. In contrast, methods first applied in 1987 to exploit axonal transport are much more accurate with more than 90% considered valid. By splitting our corpus into documents published before and after 1987, we tested for a similar signal that separates eras of experimental techniques. In agreement with the manually quantified trend, we observe an increase from 59.4 to 65.6% in the rate of connectivity statements validated in BAMS (*P* = 0.00071, hypergeometric test). We note the specificity of regions involved in the connections also increases, whereas the proportion of mapped terms is unchanged.

Connections predicted more than once might be more likely to be valid because of the effect of ‘confirmation’. This was feasible to study because, on average, each connection was predicted more than twice. The number of extracted connections per brain region (degree) provides a simple comparison with BAMS. For the 344 common brain regions, the degree vectors are strongly correlated (Pearson = 0.769, Spearman = 0.433). Counting unique predicted connections, 54.7% are in BAMS (Table 3, the value of 63.5% previously cited counts occurrences). From a recall perspective, 3.2% of BAMS connections are connected in our predictions. By thresholding our connections to those predicted at least twice, precision reaches 65.9%, whereas recall drops to 1.4% (Table 3). This accuracy is near the 67.5% precision of the hand-annotated set of connections. Precision gradually increases as the threshold increases, eventually reaching 100% for nine connections that were extracted at least 12 times. Further, we note the anatomical specificity of the connections increases with the average number of enclosing regions reaching 10.2 when thresholded at 12 occurrences. The region pairs not predicted to form connectivity relations have precision of 33.7% and recall of 9.3%. Again, this level of precision results from co-mentioned regions that are connected in BAMS, but the author is not specifying that in the sentence. Further, the higher recall value results from the much larger set of pairings (6079 compared with 1286 SLK-predicted parings). From a co-occurrence perspective, we found that brain regions that co-occur in eight or more sentences recall 1.6% of the BAMS connections at 66.4% precision. Interestingly, this naive co-occurrence-based method performs at par to the SLK method that extracts direct connectivity statements. As the threshold is increased from eight co-occurrences, precision continues to gain, suggesting a large number of co-occurring mentions can be used to predict connectivity, as well as a smaller number of more carefully analysed connectivity statements (Table 3).

**Table 3.** Aggregate connectivity results from several methods and relation sets

Relation set	Method	Threshold	Anatomical depth	Connections	Precision	Recall	F-measure
Positive annotated	Curation	1	8.7	200	67.50%	0.61%	1.22%
Negative annotated	Curation	1	8.7	1606	41.91%	3.06%	5.71%
Positive predictions	SLK	1	8.4	1286	54.70%	3.20%	6.05%
Positive predictions	SLK	2	8.4	454	65.90%	1.40%	2.74%
Positive predictions	SLK	12	10.2	9	100.00%	0.04%	0.08%
All pairings	Co-occurrence	1	8.3	6474	34.00%	10.01%	15.47%
All pairings	Co-occurrence	2	8.3	2865	44.96%	5.86%	10.37%
All pairings	Co-occurrence	8	8.2	515	66.41%	1.56%	3.04%
All pairings	Co-occurrence	16	8.4	189	71.43%	0.61%	1.22%

This table presents the analysis of the extracted binary connectivity matrices. The first two rows are from connectivity matrices derived from the 1377 annotated abstract set. The remaining rows are from the 12 557 abstract set and are split between the SLK predictions and the co-occurrence technique. The threshold column displays the required count of reported connections to be marked as connected in the matrix. Anatomical depth measures how specific the connections are by averaging the number of enclosing brain regions for each connected region.

## 4 DISCUSSION

We reported a complete system for extracting connectivity statements from biomedical abstracts. The method provides high recall of manually annotated connectivity relations described in single sentences. Precision from two separate evaluations reached 50.3% and 55.3%. By comparing with an independent source of rat connectivity, we found that precision increases with the recency and frequency of the extracted relationships.

A limitation of our work is that we did not consider the direction of connectivity, although most of the relationships we extracted have a direction described in the sentence. In addition, we did not consider negation (region A does not project to region B). Extracting this information by extracting keywords such as ‘afferent’, ‘not’ or ‘input’ will require additional work. These relationship modifiers are manually annotated in our training corpus and can be used to design more complex rules.

Our methods also did not attempt to extract the large number of relations that span multiple sentences. When these connections are taken into account, the SLK method provides only 51.7% recall of annotated connections. Application of advanced natural language-processing techniques may be necessary to bridge the sentences (e.g. anaphora resolution).

The comparison of seven previously published kernel-based approaches mirrored the previous results from the protein interaction relationship extraction domain (Tikk *et al.*, 2010). Further, we note that the SLK parser was applied to the drug–drug interaction domain with similar results (Segura-Bedmar *et al.*, 2011). Several of the kernel methods have lower performance than our simple rule-based technique. Effort spent crafting more complex rules may yield higher precision at the cost of lower recall. The top three kernel methods (SLK, all-paths graph, k-band shortest path spectrum kernel) all have similar accuracy (AUC and f-measure scores) but vary in precision and recall. This difference suggests higher performance may be achieved by combining the methods.

Our results suggest a larger set of input abstracts will yield a larger number of precise connections. The largest extension set is Medline with more than 10 million abstracts and 120 million sentences. Tikk and colleagues calculated that the SLK parser could process all of Medline in 141 days (Tikk *et al.*, 2010). A two-step process may reduce runtime and increase accuracy by first identifying abstracts with connectivity statements and then by extracting the specific connections with SLK (Poulter *et al.*, 2008).

In natural language processing, it has been observed that simple statistical models (e.g. co-occurrence) can outperform more complex models based on less data (Halevy *et al.*, 2009). Our experiments confirm this. We found that brain region pairs with many co-mentions tend to be connected. This simple technique produces a larger set of potential connections with reasonable precision. Although this will produce a larger set of results than the SLK method, it does not target connections that can be directly curated in light of experimental evidence because the co-mentions may or may not describe connectivity. Further, such co-occurrences may result from region proximity or biases (i.e. popularity) that may influence research attention both in the literature and in BAMS. However, such co-occurrence networks show valuable areas of focus when combined with comentions of genes and diseases (Hayasaka *et al.*, 2011; Voytek and Voytek, 2012).

One of the most serious challenges we encountered was in mapping extracted brain region mentions to standardized lexicons. In our previous work, we reported resolution rates of 63%, with the major limitation being gaps in the lexicons (French and Pavlidis, 2011). For the current work, the resolution rate is greatly reduced, as both brain region mentions of a connectivity relation must be mapped. It also appears that regions forming connectivity relations are harder to resolve or map, on average. For this work, we managed to double the resolution rate to the BAMS lexicon by adding synonyms. Additional work to improve the lexicons will lead to better resolution of connectivity statements, allowing validation and linking to other resources.

For our evaluation to an outside database, we focused on BAMS (Bota *et al.*, 2005). Although rat is the most frequent mentioned organism, other evaluations could compare the connectivity results with the CoCoMac (Kotter, 2004) or the Avian Brain Circuitry Database (Schrott and Kabai, 2008). Beyond evaluation, our dataset and method can provide a large set of extracted connectivity relationships for other species-specific databases.

In conclusion, we provide the first application of large-scale text mining to neuroanatomical connectivity extraction. We demonstrated that machine-learning tools designed for extraction of protein–protein interactions are generalizable to mining brain region connections. From an information retrieval perspective, our large set of predicted connections can aid neuroscientists in forming hypotheses and models. Future work will be aimed at further evaluating and disseminating the results before extending the analysis.
